# Differential analysis of ubiquitin-proteomics in skeletal muscle of Duroc pigs and Tibetan fragrant pigs

**DOI:** 10.3389/fvets.2024.1455338

**Published:** 2024-08-30

**Authors:** Meng-yu Li, Chao-cheng Li, Xin Chen, Yi-long Yao, Yu-song Han, Tao Guo, Yong-sheng Zhang, Tao Huang

**Affiliations:** ^1^Xinjiang Characteristic Livestock Breeding and Reproduction Team, College of Animal Science and Technology, Shihezi University, Shihezi, China; ^2^Animal Genetics and Breeding Group, College of Animal Science and Technology, Yangzhou University, Yangzhou, China; ^3^Kunpeng Institute of Modern Agriculture at Foshan, Foshan, Guangdong, China

**Keywords:** Duroc pigs, Tibetan fragrant pigs, skeletal muscle, ubiquitin factor, ubiquitination modification proteins

## Abstract

Understanding the differences in ubiquitination-modified proteins between Duroc pigs and Tibetan fragrant pigs is crucial for comprehending the growth and development of their skeletal muscles. In this study, skeletal muscle samples from 30-day-old Duroc pigs and Tibetan fragrant pigs were collected. Using ubiquitination 4D-Label free quantitative proteomics, we analyzed and identified ubiquitination-modified peptides, screening out 109 differentially expressed ubiquitination-modified peptides. Further enrichment analysis was conducted on the proteins associated with these differential peptides. GO analysis results indicated that the differential genes were primarily enriched in processes such as regulation of protein transport, motor activity, myosin complex, and actin cytoskeleton. KEGG pathway analysis revealed significant enrichment in pathways such as Glycolysis/Gluconeogenesis and Hippo signaling pathway. The differentially expressed key ubiquitinated proteins, including *MYL1*, *MYH3*, *TNNC2*, *TNNI1*, *MYLPF*, *MYH1*, *MYH7*, *TNNT2*, *TTN*, and *TNNC1*, were further identified. Our analysis demonstrates that these genes play significant roles in skeletal muscle protein synthesis and degradation, providing new insights into the molecular mechanisms of muscle development in Duroc pigs and Tibetan fragrant pigs, and offering theoretical support for breeding improvements in the swine industry.

## 1 Introduction

Duroc pigs, as the epitome of Western lean-type pig breeds, are renowned for their exceptional lean meat ratio and rapid growth rate in low-altitude regions (1). However, society’s extreme pursuit of lean meat ratio has led us to overlook muscle quality and the disease resistance of pigs. Tibetan fragrant pigs are local breeds in the plateau region of China. They have not only adapted to the low-oxygen environment at high altitudes, but are also favored by people for their rich intramuscular fat, delicate muscle fibers, and high content of muscle amino acids (2). It is worth mentioning that the high level of tryptophan in Tibetan fragrant pigs promotes cellular immune response and endows them with stronger disease resistance (3, 4). Despite this, Tibetan fragrant pigs also face the challenges of small body size and slow growth rate. These differences in growth rate and body size are closely related to their skeletal muscle growth and development.

Skeletal muscle accounts for about 50% of the body mass and is the primary muscle tissue responsible for maintaining normal physiological functions, energy storage, and movement control. It is mainly composed of satellite cells, immune cells, muscle fibers, the basal membrane, and nerves (5). Research has revealed that the growth potential of muscle is a crucial factor determining the production performance of animals. In piglets, significant differences in production performance primarily stem from the development status and phenotypic characteristics of their skeletal muscles (6). The development of skeletal muscles can be achieved by regulating the signaling pathways involved in protein synthesis and degradation (7). In this process, the Ubiquitin-Proteasome System (UPS) plays a vital role, influencing muscle growth and function by modulating protein stability and degradation (8, 9). The regulation of this pathway is not only essential for muscle quality and function but also an effective strategy for enhancing the production performance of piglets.

UPS refers to the process in which proteins undergo ubiquitination modifications and are subsequently degraded by the 26S proteasome (10, 11). The efficient functioning of the UPS relies on the concerted action of three key enzymes: Firstly, the ubiquitin-activating enzyme (E1), which initiates the cascade reaction by activating the ubiquitin molecules in the first step. Following that is the ubiquitin-conjugating enzyme (E2), which serves as an intermediary, transferring the activated ubiquitin molecules to the correct target proteins. Lastly, the ubiquitin ligase (E3), with its high substrate specificity, identifies the target proteins and catalyzes the formation of covalent bonds between the ubiquitin molecules and the target proteins. This coherent process ensures precise protein degradation, maintaining the stability of the intracellular environment and regulating protein structure and function (12). Ubiquitination is the process by which ubiquitin molecules covalently attach to target proteins, affecting their stability, function, and subcellular localization. Ubiquitination primarily functions through two forms: monoubiquitination and polyubiquitination. Monoubiquitination involves the attachment of a single ubiquitin molecule to multiple distinct lysine residues on the target protein. It primarily participates in various cellular processes such as endocytosis, chromatin remodeling, lysosomal targeting, and meiosis. In contrast, polyubiquitination involves the concatenation of multiple ubiquitin molecules to form a chain, which is then linked to a single lysine residue on the target protein through this chain-like structure. This form of ubiquitination plays a vital role in essential physiological processes such as protein degradation within cells, immune signal transduction, and DNA repair (13, 14).

Ubiquitination influences the metabolic properties and growth of muscle fibers by regulating the degradation of key metabolic enzymes and structural proteins. The postnatal muscle growth potential in pigs primarily relies on the total number and hypertrophy degree of muscle fibers within skeletal muscle tissue. A greater abundance of muscle fibers, coupled with a higher degree of hypertrophy, enables piglets to exhibit superior lean meat growth capabilities, thereby facilitating a quicker attainment of the desired market weight standards (15, 16). Within the first 30 days of life, pigs experience a significant increase in the total number of muscle fibers, which subsequently plateaus after this age (17). During this growth phase, genes that regulate muscle fiber development and growth, such as Myostatin (MSTN) and Myogenic Differentiation Factor (MyoD), function under the influence of ubiquitination modifications, with their expression peaking at around 30 days of age (18).

Skeletal muscle fibers are categorized into Type I, Type IIa, and Type IIb based on their metabolic properties and contraction speeds (19). Muscles contain a number of different muscle fiber types, but are dominated by one type of muscle fiber (20). In the skeletal muscles of Tibetan fragrant pigs, oxidative/slow/type I muscle fibers predominate, utilizing aerobic oxidation metabolism for energy supply (21). In contrast, the skeletal muscles of Duroc pigs are dominated by Glycolytic/fast/type IIb muscle fibers, with more active glycolytic metabolism and a greater tendency to acquire energy through the glycolytic pathway (22). This variation in muscle fiber types not only determines the muscular quality of different pig breeds but also significantly impacts their growth potential and meat characteristics (23). Nevertheless, comparative studies on ubiquitination modifications in the skeletal muscle tissues of Duroc pigsand Tibetan fragrant pigs are still relatively scarce. Delving deeper into these differences may help us better comprehend the molecular mechanisms of muscle growth and how we can improve meat quality and increase production efficiency by regulating biochemical processes such as ubiquitination.

This study aims to investigate the differences in ubiquitination modifications during myogenesis in 30-day-old Duroc pigs and Tibetan fragrant pigs. We will identify and quantify ubiquitination sites and perform GO and KEGG pathway enrichment analyses, followed by constructing a PPI network. By analyzing the ubiquitin-tagged protein interaction networks involved in muscle metabolism in different pig breeds, this research provides a basis for revealing the molecular mechanisms underlying muscle growth and development.

## 2 Materials and methods

### 2.1 Sample collection

Sample animals were provided by a farm in Shihezi City. The study subjects were three each of Duroc pigs and Tibetan fragrant pigs at 30 days of age. The pigs were anaesthetized separately and slaughtered, and specimens were taken from the tibialis anterior muscle of the hind limb of each pig. The Samples from the left and right legs were mixed and preserved.

### 2.2 Protein extraction and digestion

The preserved muscle samples were lysed using a urea buffer (8 M/L Urea, 100 mM/L Tris/HCl, pH 8.5) to extract proteins. Protein concentration was determined using the Bradford protein assay kit. A 12.5% SDS-PAGE gel was prepared, and 20 μg of extracted protein was mixed with 5× loading buffer, boiled for 5 min, and then subjected to electrophoresis for 90 min. The protein bands were stained with Coomassie Brilliant Blue R-250 for visualization.

The samples were mixed with DTT (10 mM/L) at 600 rpm at room temperature (37°C) for 90 min. After cooling, 50 mM/L IAA was added and the mixture was incubated in the dark for 30 min. The urea concentrate was then diluted to 2 M with Tris HCl (pH 8.0). Trypsin was added at a ratio of 1:50 (trypsin) and the mixture was incubated overnight (15–18 h) at room temperature (37°C). Following digestion, 0.1% TFA was added, and the solution was adjusted to pH ≤ 3 with 10% TFA. The resulting digested peptides were desalted using an Empore TMSPE C18 cartridge and then lyophilized for subsequent use.

### 2.3 Enrichment of modified peptides

To enrich modified peptides, 1.4 mL of pre-chilled IAP buffer was mixed with the sample for dissolution. Subsequently, processed Anti-K-ε-GG antibody beads [PTMScan Ubiquitin Remnant Motif (K-ε-GG)] were added to the mixture, followed by an incubation at 4°C for 90 min. Afterward, centrifugation was performed at 2000 × G for 30 s to remove the supernatant, which was then washed three times with 1 mL of pre-chilled IAP buffer. This washing step was repeated using pre-chilled distilled water three times. Next, 40 μL of 0.15% TFA was added and incubated at room temperature for 10 min, followed by an additional 40 μL of 0.15% TFA. Finally, centrifugation at 2000 × G for 30 s was conducted to collect the supernatant, which was further desalted using C18 STAGE Tips.

### 2.4 LC–MS/MS analysis

We performed liquid chromatography–tandem mass spectrometry (LC–MS/MS) analysis using the timsTOF Pro mass spectrometer coupled with Nanoelute (Bruker Daltonics) over a continuous 60-min period. A custom-made C18 reverse-phase analytical column (25 cm length, 75 μm inner diameter, C18 material) was utilized. The chromatographic separation employed two mobile phases: Buffer A (0.1% formic acid) and Buffer B (84% acetonitrile, 0.1% formic acid), with a gradient elution method. The flow rate was set at 300 nL/min. The mass spectrometer operated in positive ion mode with a mass range of m/z 100–1700. Ion mobility was set within the range of 1/k0 from 0.6 to 1.6. During data acquisition, PASEF MS/MS analysis was conducted with settings of 1.5 k target intensity, 2,500 threshold, and 10 PASEF MS/MS cycles. To enhance accuracy and efficiency, we activated the dynamic exclusion feature with a release time of 0.4 min.

### 2.5 Identification and quantitation of modified proteins

We will merge the raw mass spectrometry (MS) data collected from each sample into a unified dataset. Subsequently, we will use the bioinformatics software MaxQuant for comprehensive protein identification. Through MaxQuant, we can accurately identify protein species present in the samples, perform quantitative analysis, and calculate protein expression levels. This analysis will reveal differences in protein expression levels between samples of Duroc pigs and Tibetan fragrant pigs’ skeletal muscles, providing crucial insights into the ubiquitinated proteomic characteristics of these tissues.

### 2.6 Bioinformatics analysis

#### 2.6.1 Cluster analysis of modified peptides

Hierarchical clustering analysis was performed using Cluster 3.0 and Java Treeview software tools. During clustering, the Euclidean distance algorithm was selected as the similarity metric, and the average linkage clustering method was employed for clustering operations, using the centroids of observational data as references. To visually present the clustering results comprehensively, in addition to generating dendrogram trees, heat maps were utilized as visual aids. The dendrogram provided hierarchical relationships among samples, while the heat map allowed for intuitive observation of sample expression profiles across various features. This approach further elucidated the differences and similarities in the ubiquitin proteomics profiles of skeletal muscles between Duroc pigs and Tibetan fragrant pigs.

#### 2.6.2 Subcellular localization

Using the multi-class Support Vector Machine (SVM) within the CELLO classification system, we conducted precise predictions of protein subcellular localization. This method allowed us to effectively infer the specific locations of proteins within cells, providing crucial information and guidance for our research. SVM, known for its robust machine learning capabilities, demonstrated excellent performance and accuracy in predicting protein subcellular localization, thus offering reliable support and foundations for our study.

#### 2.6.3 Protein domain analysis

We utilized InterProScan software to query the Pfam database within the InterPro member database, enabling the identification and analysis of protein domain features within protein sequences. This process allowed us to gain a comprehensive understanding of protein structure and function, identifying functional domains and further inferring their potential biological functions and involvement in metabolic pathways.

#### 2.6.4 GO and KEGG analysis

We used the NCBI BLAST+ client software (NCBI-BLAST-2.2.28 + −win32.exe) and InterProScan tool to search for homologous sequences of differentially expressed protein sequences, aiming to identify known sequences related to our studied proteins. Subsequently, we utilized Blast2GO for GO term mapping and sequence annotation. Finally, we employed R scripts to visualize the GO annotation results, facilitating a more intuitive presentation of the protein functional information.

In addition, we compared the studied proteins with the KEGG database to retrieve homology identification information of the proteins in KEGG. By mapping this information to relevant pathways in KEGG, we gained a deeper understanding of the biological functions of these proteins and the metabolic pathways they are involved in.

#### 2.6.5 Enrichment analysis

We performed enrichment analysis on all quantified proteins using Fisher’s exact test, and adjusted the *p*-values using the Benjamini-Hochberg method to control the false discovery rate from multiple comparisons. During this process, we retained only functional categories and pathways with corrected *p*-values *p* < 0.05 as significant results. This approach effectively filtered out functional features and biological pathways that showed significant differences between samples.

#### 2.6.6 Protein–protein interaction network analysis

We utilized gene symbols or the STRING software to retrieve protein–protein interaction (PPI) information from molecular interaction databases for the studied proteins. The search results were downloaded in XGMML format and imported into Cytoscape software for visualization and analysis.

## 3 Results

### 3.1 Ubiquitination identification and quantitative analysis

**Figure 1:**
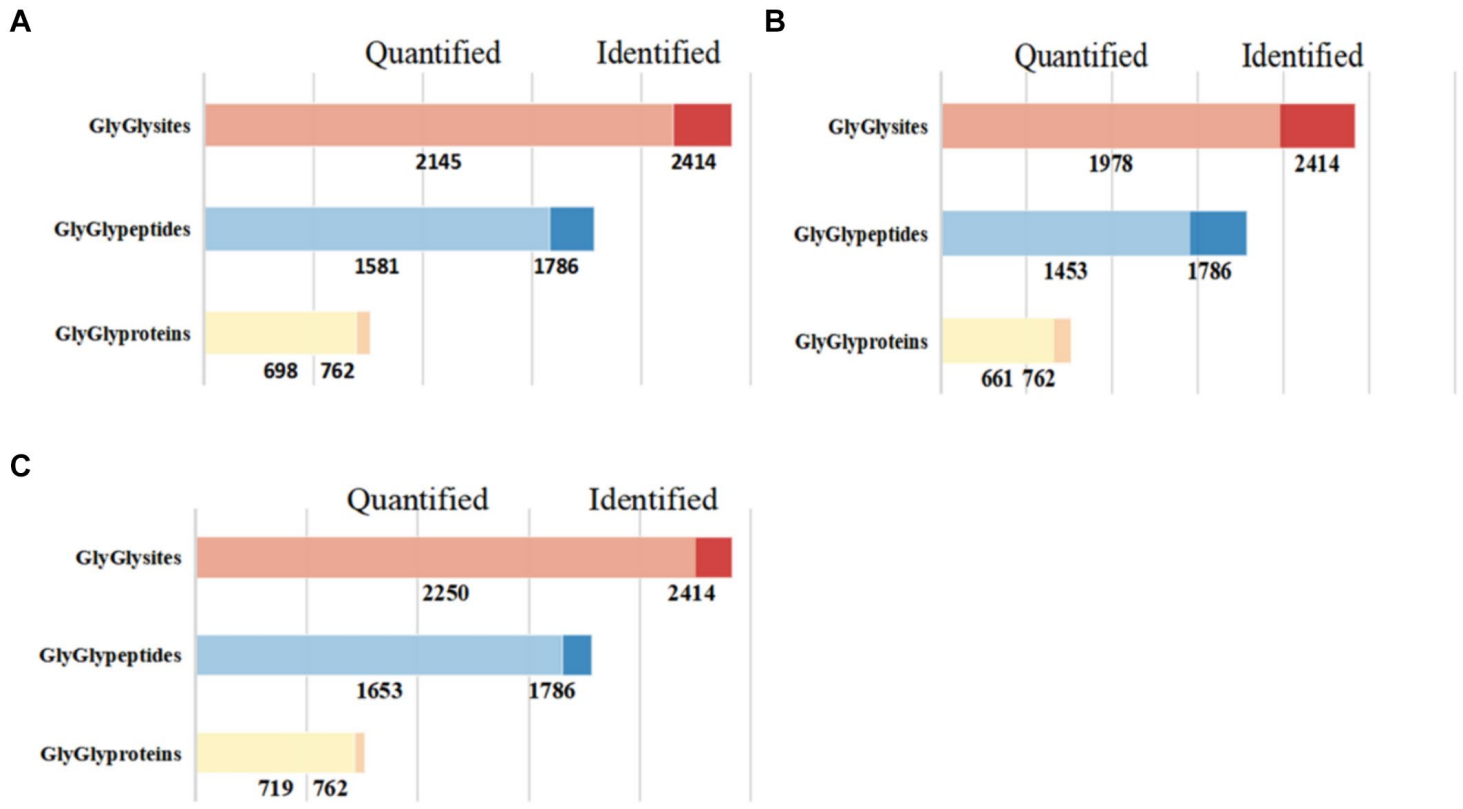
Quantitative results of ubiquitinated proteins in skeletal muscle of Duroc pigs and Tibetan fragrant pigs. **(A)** Shows the bar chart of identification and quantitative statistics of ubiquitinated proteins, peptides and sites in Duroc pig; **(B)** is the bar chart of identification and quantitative statistics of ubiquitinated proteins, peptides and sites in Tibetan fragrant pig; **(C)** represents the overlap of identification and quantitative statistics of ubiquitinated proteins, peptides and sites between Duroc pigs and Tibetan fragrant pigs.

In this study, we successfully identified 762 ubiquitinated proteins, 1,786 ubiquitinated peptides, and 2,414 ubiquitination sites (Figure 1). Among these identified results, 1,653 ubiquitinated peptides and 2,250 ubiquitination sites were quantifiable, and they were distributed across 719 modified proteins.

### 3.2 Distribution of ubiquitination modification sites

**Figure 2:**
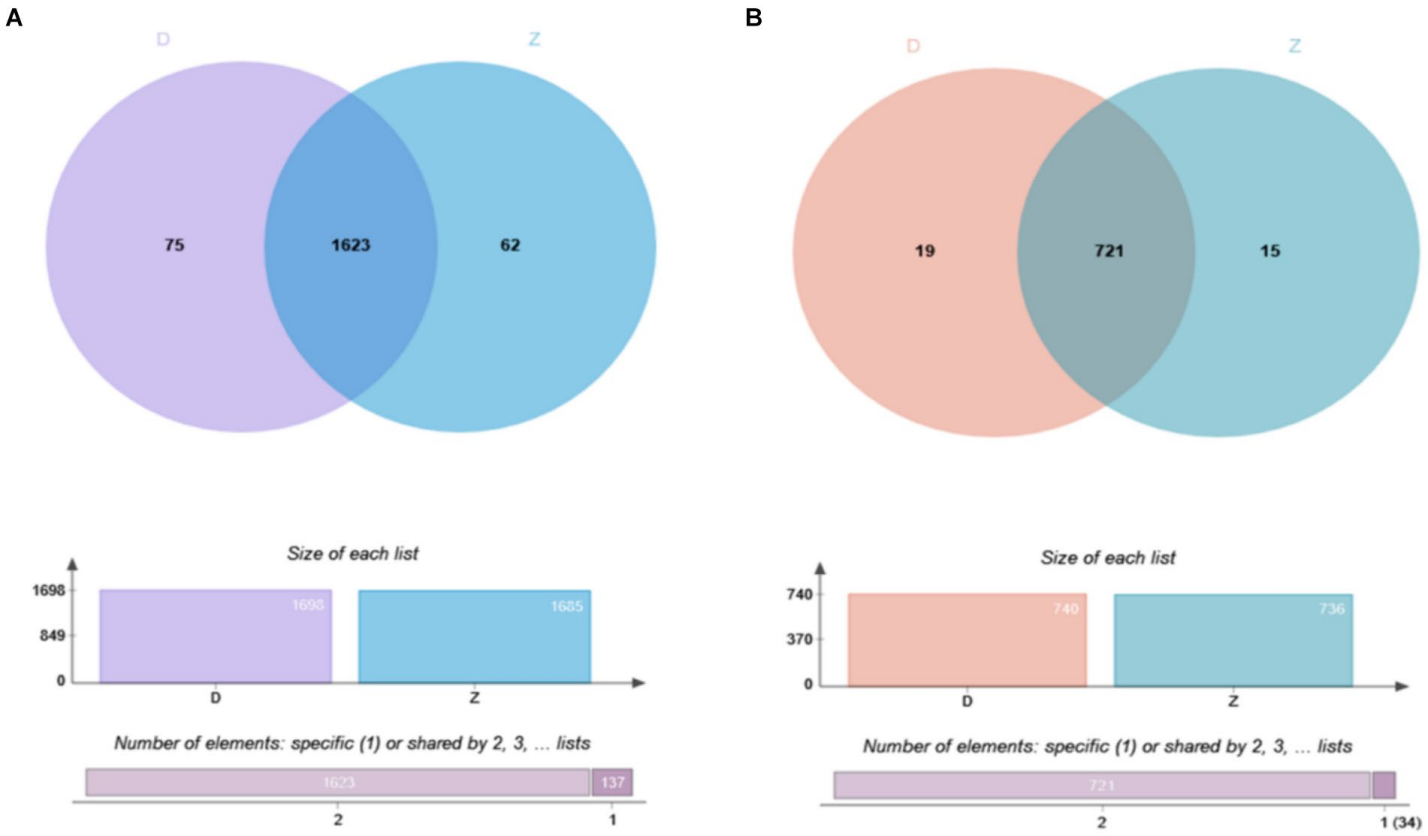
Overlapping results of ubiquitination modification identification of samples are shown as follows: **(A)** Overlap of ubiquitinated modified peptides; **(B)** overlap of ubiquitinated modified proteins.

Using a Venn diagram to analyze the identification results, we explored the overlap of ubiquitination modifications between samples. At the level of ubiquitinated peptides (Figure 2A), we found that 1,623 peptides showed significant overlap, while 62 peptides were unique to the Tibetan fragrant pigs samples, and 75 peptides were unique to the Duroc pig samples. These unique peptides reveal distinct characteristics of ubiquitination modifications in the two samples. At the level of ubiquitinated proteins (Figure 2B), 721 proteins were common between the samples, with 15 proteins unique to the Tibetan fragrant pigs samples and 19 proteins unique to the Duroc pig samples. These unique proteins highlight the subtle differences in ubiquitination modifications between the two samples.

### 3.3 Differential expression analysis

**Figure 3:**
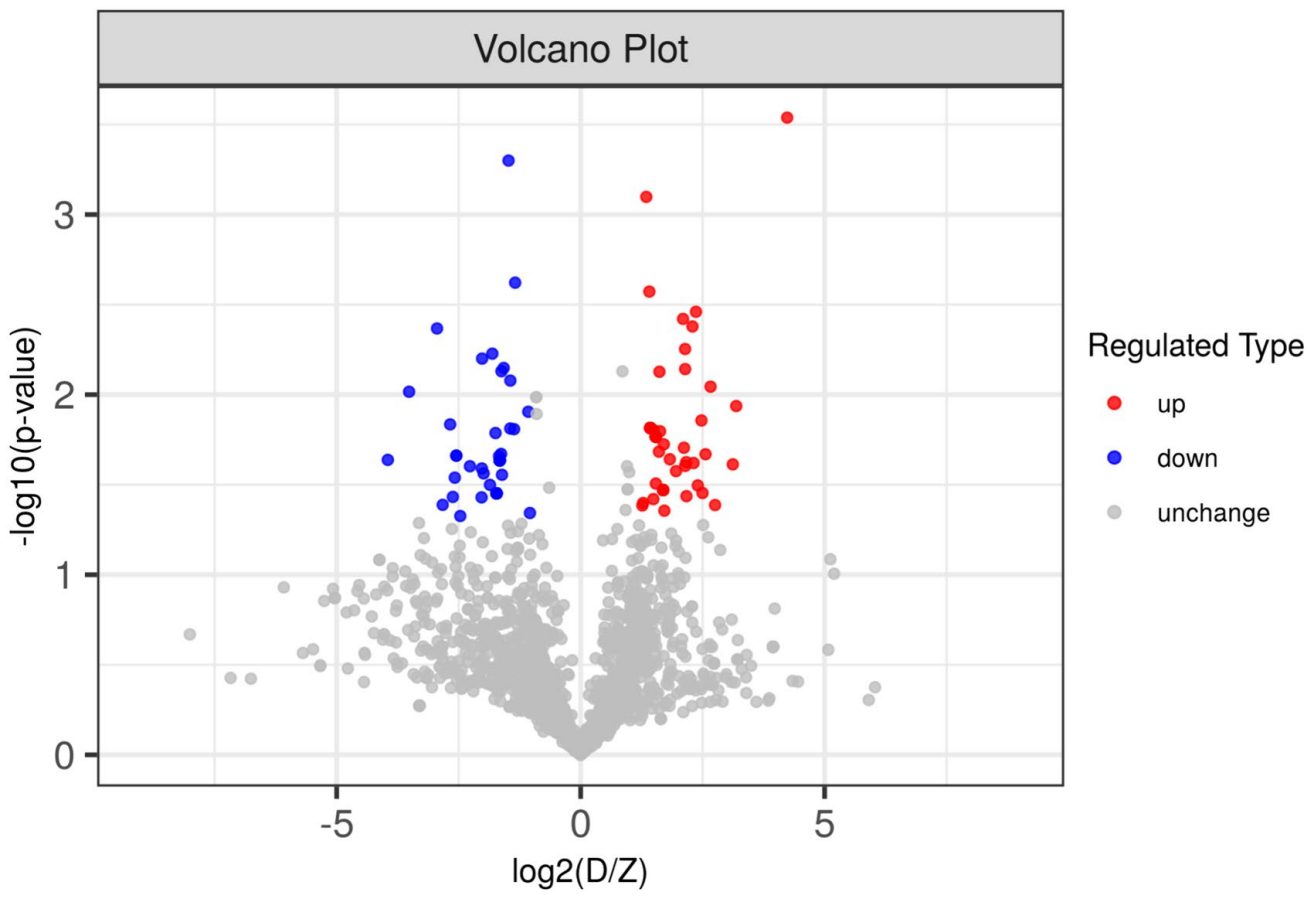
Differences in ubiquitinated modified peptides between Duroc pigs and Tibetan fragrant pigs. Up-regulated ubiquitinated modified peptides are shown in red, down-regulated ubiquitinated modified peptides are shown in blue, and ubiquitinated peptides with no difference change are shown in gray.

**Figure 4:**
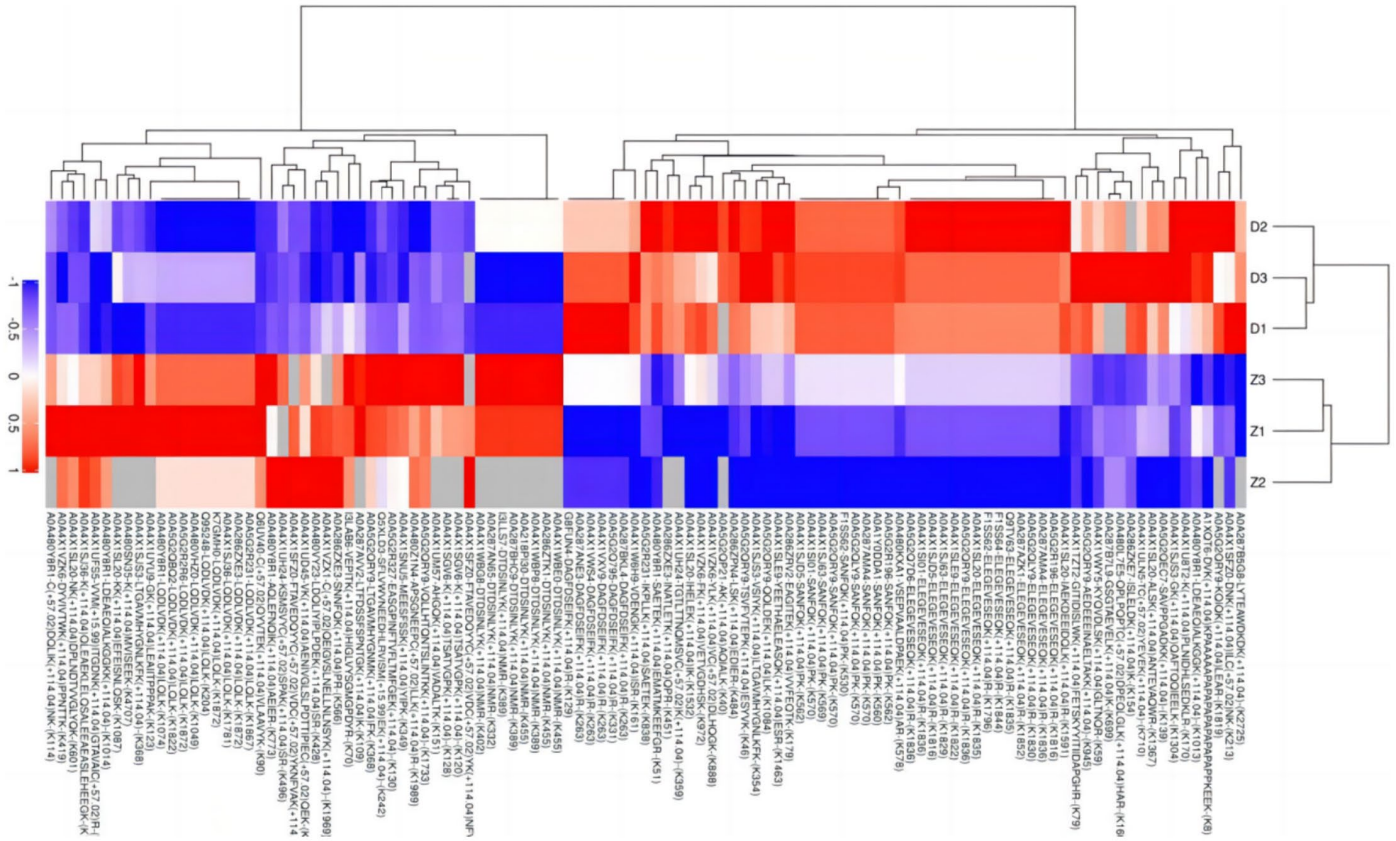
Tree heat map of the results of cluster analysis of differentially expressed ubiquitinated peptides in Duroc pigs and Tibetan fragrant pigs. The ordinate represents the sample information and the abscissa represents the significantly differentially expressed peptides. Different colors are shown in the heat map, where red represents significantly up-regulated peptides, blue represents significantly down-regulated peptides, and gray represents peptide-free quantitative information.

Due to the inconsistent expression changes of ubiquitination modification sites on proteins, it is challenging to conduct a comprehensive quantitative analysis. To identify differentially expressed modified peptides, we filtered the data for ubiquitinated peptides with significant differences (|FC| ≥ 2 and *p* < 0.05). Among the 1,653 ubiquitinated peptides, a total of 109 differentially expressed ubiquitinated peptides were identified, with 62 ubiquitinated peptides significantly upregulated and 47 ubiquitinated peptides significantly downregulated (Figure 3). Clustering analysis of these differentially expressed ubiquitinated peptides revealed significant differences in the ubiquitination profiles between the skeletal muscles of Duroc pigs and Tibetan fragrant pigs (Figure 4). This differential expression highlights the distinct ubiquitination modifications that may contribute to the unique muscle growth and development characteristics of each pig breed.

### 3.4 Subcellular localization analysis

**Figure 5:**
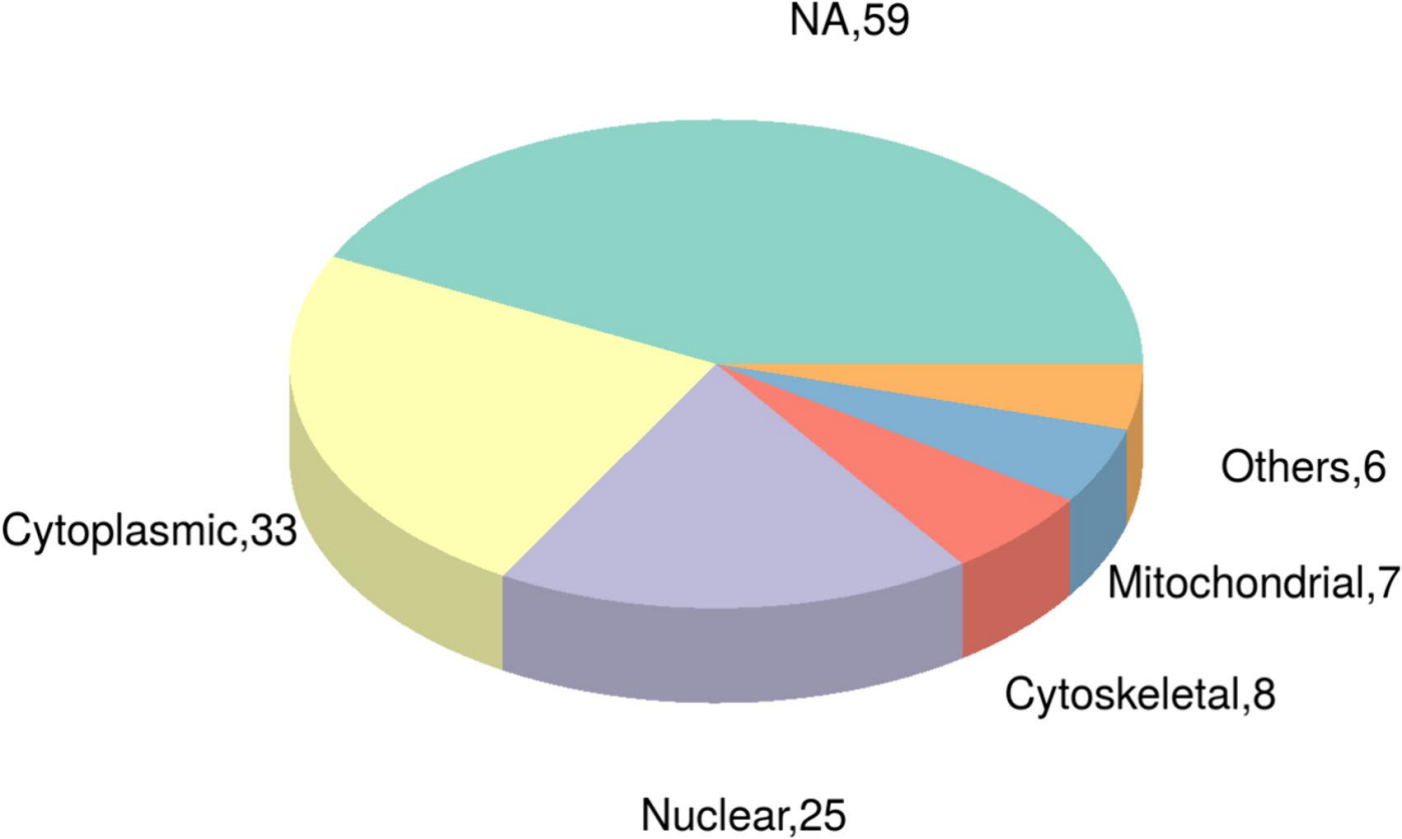
Pie chart of subcellular localization of proteins belonging to differentially expressed modified peptides in Duroc pigs and Tibetan fragrant pigs.

Utilizing the CELLO subcellular structure prediction software, we conducted subcellular localization analysis of all differentially expressed modified peptide segments corresponding to proteins. Through clear visualization using pie charts (Figure 5), we demonstrated the quantitative distribution of modified proteins within various cellular organelles. The results revealed 33 modified proteins distributed in the cytoplasm, 25 in the nucleus, 8 in the cytoskeleton, 7 in the mitochondria, and 6 in other extracellular and membrane locations. Additionally, 59 proteins corresponding to differentially expressed modified peptide segments exhibited uncertain localization within the cell, providing crucial data support for subsequent cellular studies.

### 3.5 Protein domain analysis

**Figure 6:**
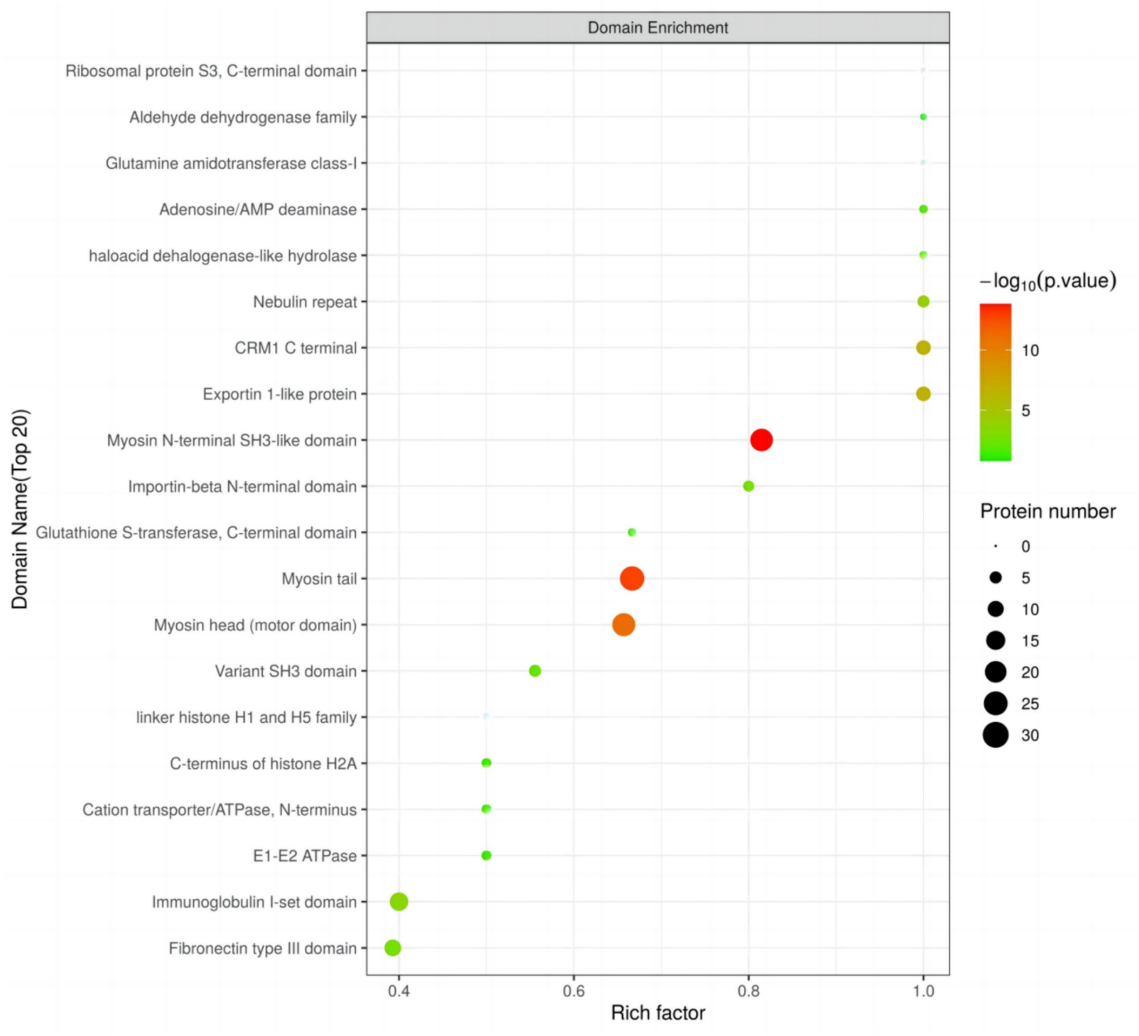
Domain enrichment analysis of Duroc pigs and Tibetan fragrant pigs.

Using Fisher’s exact test, we evaluated the significance level of protein enrichment in specific domains (Figure 6), providing a detailed analysis of the enrichment characteristics of proteins belonging to differentially expressed modified peptide segments at the domain level. Among the top 20 protein domains associated with differentially expressed modified peptide segments, a total of 12 domains exhibited significant enrichment. Notably, the Myosin N-terminal SH3-like domain, Myosin tail, Myosin head (motor domain), Exportin 1-like protein, CRM1 C terminal, Nebulin repeat, Immunoglobulin I-set domain, Fibronectin type III domain, Importin-beta N-terminal domain, and Variant SH3 domain showed extremely significant enrichment. Additionally, the haloacid dehalogenase-like hydrolase and Adenosine/AMP deaminase domains demonstrated significant enrichment.

### 3.6 Gene ontology (GO) functional analysis

**Figure 7:**
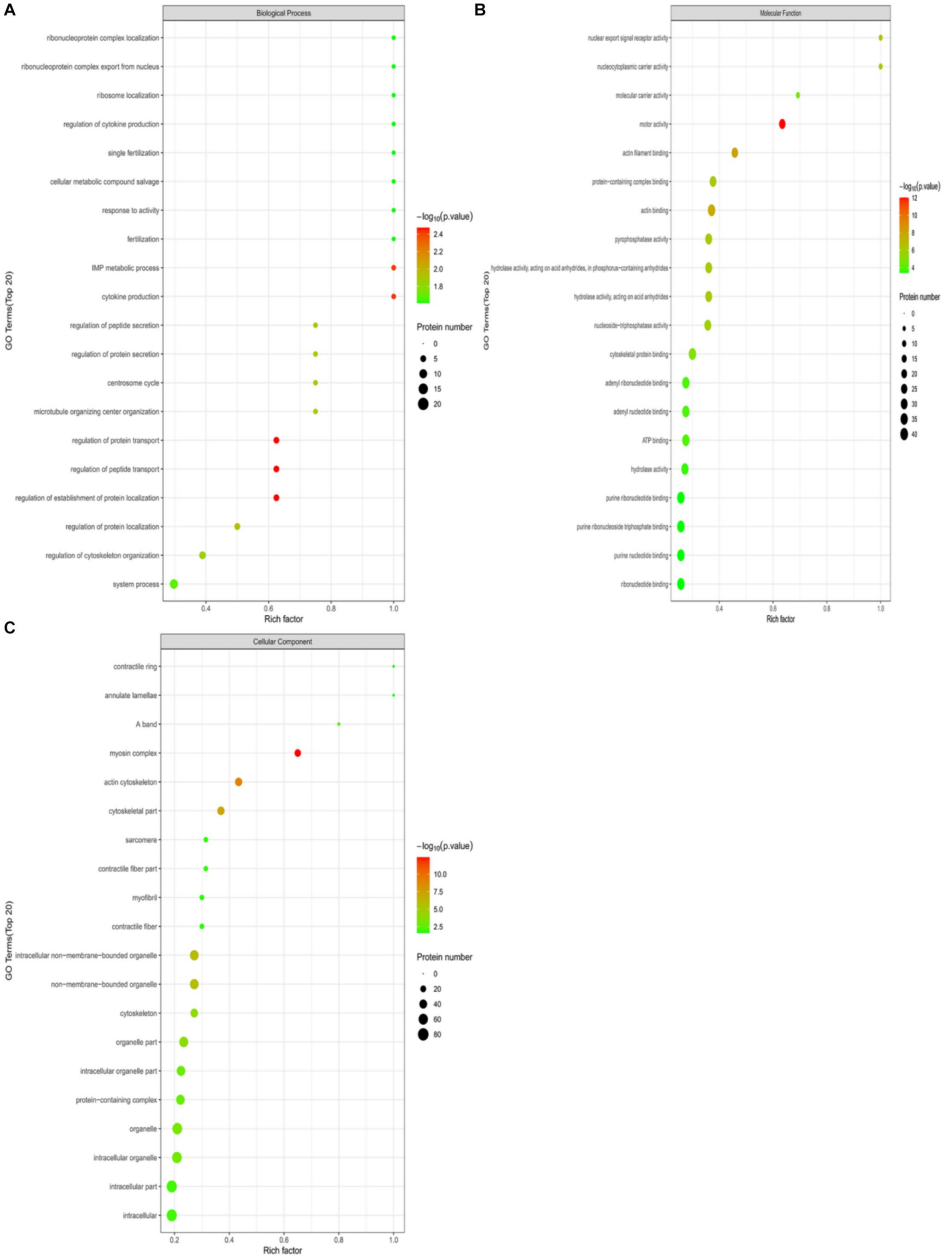
GO functional enrichment bubble plots for Duroc pigs and Tibetan fragrant pigs. **(A)** Under the classification of Biological Process (BP); **(B)** under Molecular Function (MF); **(C)** cellular Component (CC). Bubble color represents the significance of enriched GO functional classification, that is, the *p*-value is calculated based on Fisher’s exact test, the color gradient represents the magnitude of *p*-value (take –log10), the closer the color is to red, the smaller the *p*-value (*p* < 0.05), and the higher the significance of the corresponding GO functional category enrichment.

Perform GO functional annotation and enrichment analysis on differentially expressed ubiquitinated peptide-related proteins to evaluate the significance of protein enrichment under specific GO functional terms. In the Biological Process (BP) category, significant changes were observed in processes such as regulation of protein transport, regulation of establishment of protein localization, regulation of peptide transport, cytokine production, and IMP metabolic process (Figure 7A). At the Molecular Function (MF) level, significant changes were noted in functions such as motor activity, actin filament binding, actin binding, nuclear export signal receptor activity, and nucleocytoplasmic carrier activity (Figure 7B). Regarding Cellular Component (CC), significant changes were observed in components such as myosin complex, actin cytoskeleton, cytoskeletal part, non-membrane-bounded organelle, and intracellular non-membrane-bounded organelle (Figure 7C).

To demonstrate the hierarchical relationship of enriched Gene Ontology (GO) terms associated with differential ubiquitinated peptide-protein pairs, we employed top Gene Ontology Directed Acyclic Graphs (DAGs). In this graph, functional scopes are defined from top to bottom, where branches represent inclusive relationships leading to more specific functional categories. We selected the top 10 most enriched terms as main nodes, identified by squares. Connections between related GO terms are depicted through inclusion relationships, represented by circles (see Supplementary Figures S1–S3 for details).

### 3.7 KEGG pathway annotation

In this study, we annotated the KEGG pathway for differentially expressed ubiquitinated peptide-protein pairs and counted the number of associated proteins. The results revealed that the most frequently involved KEGG pathways included Motor proteins, Glycolysis/Gluconeogenesis, Arginine and proline metabolism, Hippo signaling pathway, and Neutrophil extracellular trap formation.

**Figure 8:**
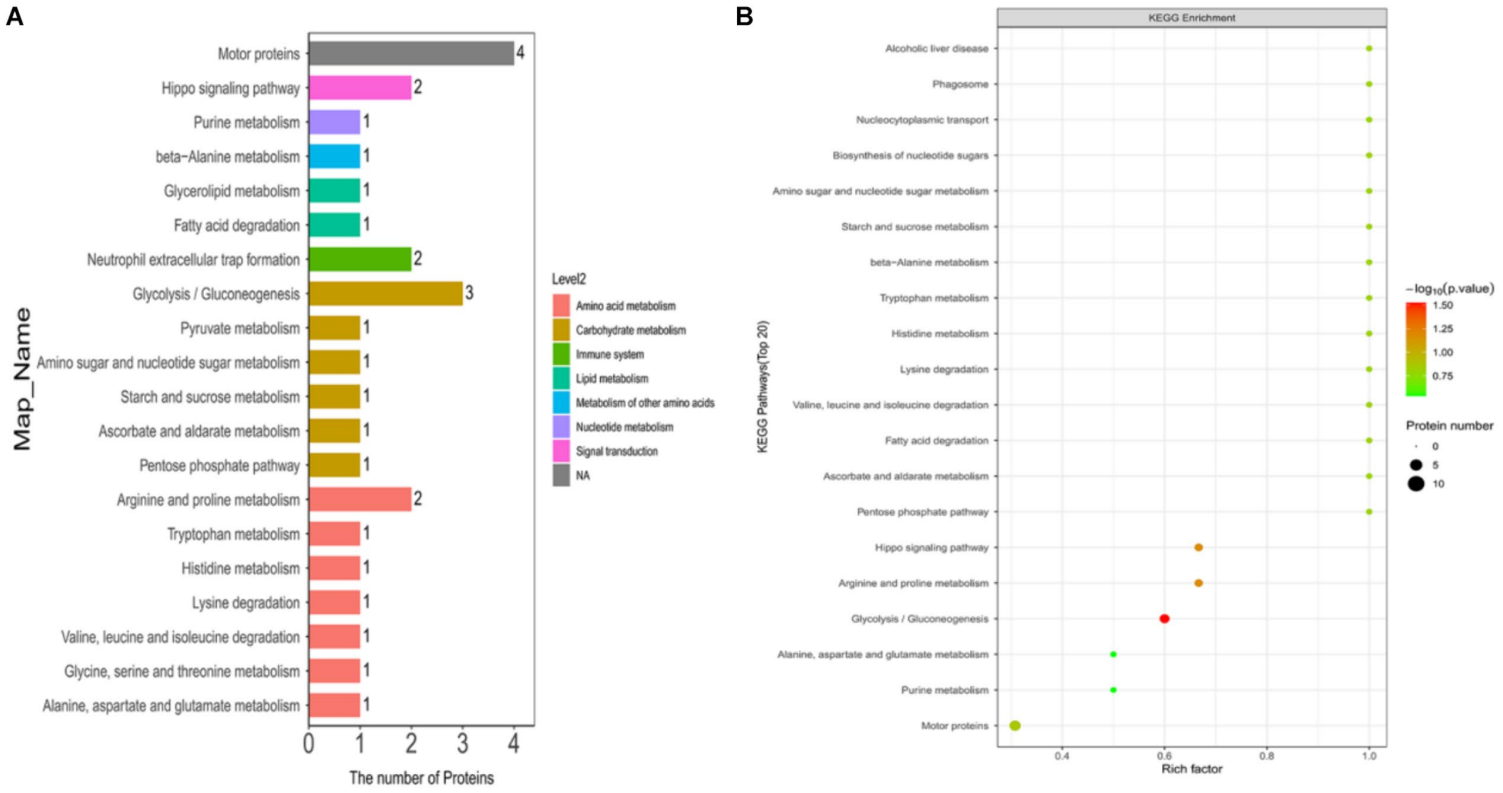
KEGG pathway maps of differentially expressed modified peptide proteins in Duroc pigs and Tibetan fragrant pigs. **(A)** KEGG pathway annotations and attribution bar graphs. The different colors represent the seven branches of the metabolic pathways of the KEGG. **(B)** KEGG enrichment bubble map (Top20). Bubble color represents the significance of enriched GO functional classification, that is, the *p* value is calculated based on Fisher’s exact test. The color gradient represents the magnitude of *p* value (take –log10), the closer the color is to red, the smaller the *p* value (*p* < 0.05), and the higher the significance of metabolic pathway enrichment.

In this study, we explored the enrichment of differentially expressed ubiquitinated peptide segments in different metabolic pathways from seven aspects: Cellular Processes, Environmental Information Processing, Genetic Information Processing, Human Diseases, Metabolism, Organismal Systems, and Drug Development (Figure 8A).

**Figure 9:**
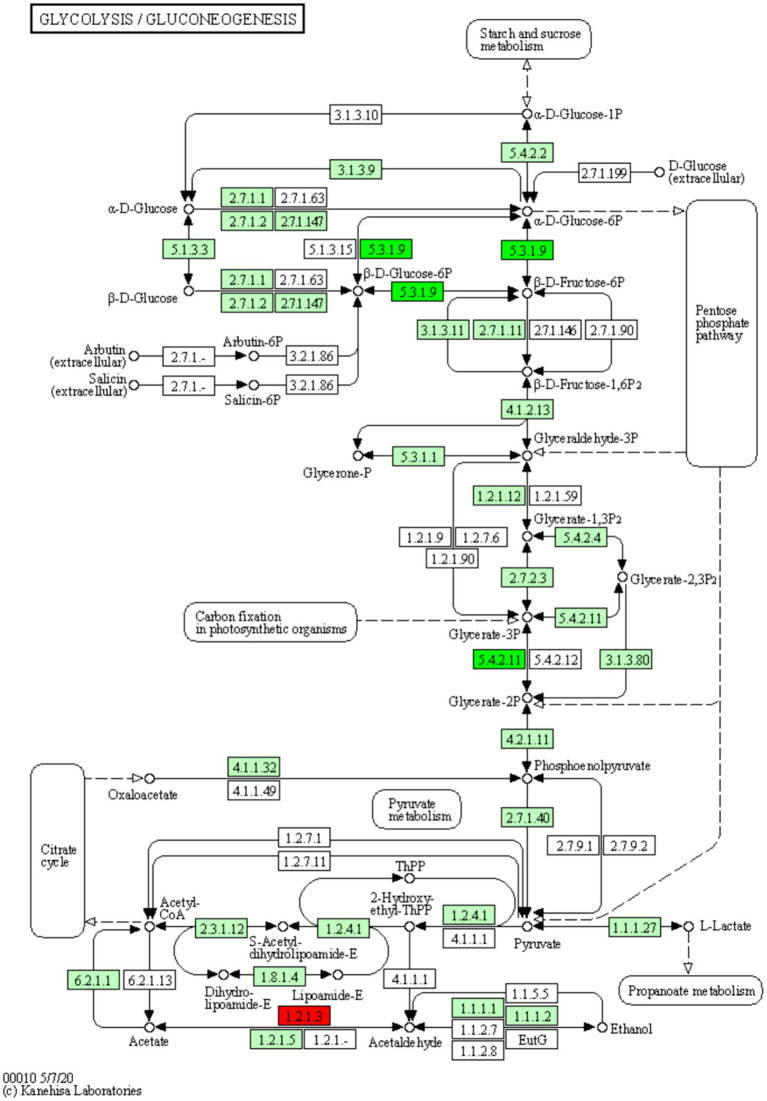
Diagram of the glycolysis/glycolysis pathway of the differentially expressed protein KEGG. The red boxes in the figure indicate that the modified peptides in the modified proteins that are differentially expressed are all up-regulated, and the green boxes indicate that the modified peptides in the modified proteins that are differentially expressed are all down-regulated. Small circles represent small molecule metabolites, and large circles represent other pathways. Light green boxes are species-specific proteins.

The pathways enriched for differentially expressed proteins were determined by comparing the KEGG annotations of the identified proteins with those of proteins associated with differentially expressed ubiquitinated peptide segments and assessing the significance of the disparity (*p* < 0.05). The analysis results indicate significant changes in key pathways such as Glycolysis/Gluconeogenesis, Hippo signaling pathway, and Arginine and proline metabolism (Figure 8B). These pathways play crucial roles in cellular biology; for example, Glycolysis/Gluconeogenesis is a pivotal process in energy metabolism, essential for maintaining energy balance and cellular survival. The Hippo signaling pathway is a conserved signaling cascade involved in regulating cell proliferation, apoptosis, and organ size. Meanwhile, Arginine and proline metabolism participate in protein synthesis as well as nitrogen balance and transport. These pathway alterations reveal the potential roles of differentially expressed ubiquitinated proteins in specific biological processes. The information of differentially expressed ubiquitinated proteins in the Glycolysis/Gluconeogenesis pathway and their positions within the pathway are depicted in the Figure 9.

### 3.8 Protein–protein interaction network analysis

**Figure 10:**
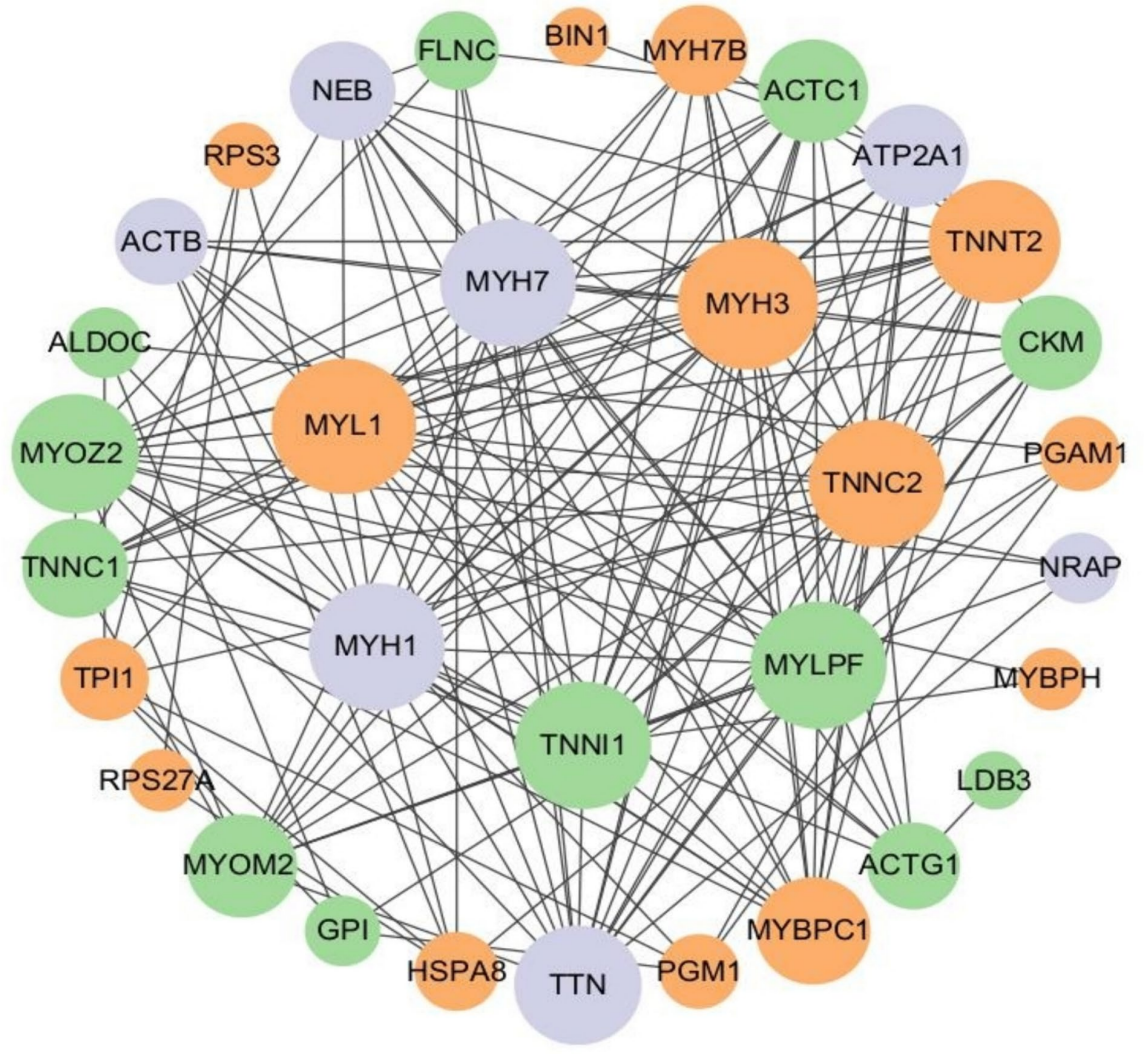
Protein interaction network of ubiquitinated peptides differentially expressed in Duroc pigs and Tibetan fragrant pigs. The circled nodes in the figure indicate the proteins to which the differentially expressed modified peptides belong, and the lines indicate protein–protein interactions. The color of the circles indicates the difference in expression of the modified peptides on the protein (green marks down, orange marks up, gray marks if there are multiple modified peptides on the protein with opposite upward and downward trends), and the size of the circles indicates protein connectivity (i.e., the number of proteins that directly interact with a protein).

In this study, a protein–protein interaction network diagram was constructed using CytoScape software, based on the protein interaction data from the STRING databases, for the proteins corresponding to differentially expressed ubiquitinated peptide segments (Figure 10). Analysis revealed that genes such as *MYL1*, *MYH3*, *TNNC2*, *TNNI1*, *MYLPF*, *MYH1*, *MYH7*, *TNNT2*, *TTN*, and *TNNC1* play indispensable roles in protein pathways. Specifically, *MYL1*, *MYH3*, *TNNC2*, and *TNNT2* were upregulated in protein pathways, while *TNNI1*, *MYLPF*, and *TNNC1* were downregulated. Moreover, multiple ubiquitinated peptide segments were identified on the *MYH1*, *MYH7*, and *TTN* proteins, with both upregulated and downregulated expressions.

## 4 Discussion

Based on the experimental results, we observed significant differences in the ubiquitin proteomic profiles between Duroc pigs and Tibetan fragrant pigs skeletal muscles. These notable variations in protein expression play crucial roles in key biological processes such as muscle development and metabolic regulation. Through further analysis of protein functionality and regulatory networks, we can gain a deeper understanding of the specific mechanisms through which these differentially expressed proteins contribute to skeletal muscle development and metabolism in pigs, thus advancing research in skeletal muscle ubiquitination.

Conducting conservative motif analysis on proteins aids in predicting their functions and gaining deeper insights into the mechanisms of ubiquitination-modified proteins. However, in this experiment, no significant conservative motifs were identified when performing conservative motif analysis on proteins belonging to differentially expressed ubiquitination-modified peptide segments. This could be attributed to the diversity and specificity of ubiquitination modifications, which prevent the formation of prominent conservative motifs across different proteins.

The results of the present study show significant differences in the Glycolysis/Gluconeogenesis pathway associated with differentially expressed ubiquitinated peptides. Glycolysis and Gluconeogenesis are central processes that regulate cellular energy and glucose levels, and their close interaction ensures efficient energy use and stable blood glucose levels. Glycolysis is the catabolic process that breaks down glucose into energy and intermediates required for biosynthesis. Conversely, Gluconeogenesis is the key gluconeogenic process that maintains blood glucose levels, especially during fasting. Although these processes share a number of enzymes, they are not only opposites, but also complex systems of coordination and regulation. Pyruvate kinase (PK) and phosphoenolpyruvate carboxykinase (PEPCK) have been found to be the key enzymes of Glycolysis and Gluconeogenesis, catalyzing the final and first steps of their respective processes, respectively. The activities of both enzymes are regulated by lysine acetylation. Specifically, PKM (a muscle form of PK) undergoes an acetylation modification at the lysine 305 site, which reduces its enzymatic activity and triggers chaperone-mediated autophagy (CMA, Chaperone-mediated Autophagy), which ultimately directs PKM into the lysosome for degradation. On the other hand, for PEPCK, its acetylation regulation mechanism is closely related to the HECT (homologous to E6AP C-terminus) structural domain family of the E3 ubiquitin ligase family. In particular, this family member uBR5 (ubiquitin protein ligase E3 component n-recognin 5) drives the ubiquitination tagging of PEPCK, which, subsequently, is recognized and degraded by the proteasome (24). In the present study, we found significant disparity in glycolysis/glycolysis pathway between Duroc and Tibetan fragrant pigs. This result is consistent with previous findings. Ubiquitination, as an important regulatory mechanism, plays a key role in the contraction and energy supply of muscle tissue by affecting key enzymes of Glycolysis and Gluconeogenesis. Studies have shown that energy metabolism in fast muscle fibers is mainly dependent on the Glycolysis pathway (25). Brocks’ study further revealed that the proportion of glycolytic muscle fibers increased in pig breeds with higher lean body mass and the diameter of these muscle fibers increased accordingly (26). These results suggest that the muscle tissue of Duroc pigs contains a higher proportion of glycolytic muscle fibers. During ubiquitination modification, the Glycolysis pathway is preferred to obtain the required energy.

In this study, the genes coding for the proteins belonging to the differentially expressed ubiquitination modification peptides were identified by PPI network analysis, including *MYL1*, *MYH3*, *TNNC2*, *TNNI1*, *MYLPF*, *MYH1*, *MYH7*, *TNNT2*, *TTN*, and *TNNC1*. These genes play crucial roles in the structure and function of muscle tissue. Actin, myosin, troponin, and tropomyosin collectively form muscle fiber tissue (27, 28). Myosin is a vital component of muscle tissue, and its encoding function is mainly regulated by the *MYH* gene family. This family encodes various components including myosin heavy chains (MHC), regulatory light chains (RLC), and essential light chains (ELC) (29). Among these genes, *MYLPF* (Fast skeletal muscle myosin regulatory light chain 2), a phosphorylatable fast skeletal muscle gene, commonly known as *HUMMLC2B*. It is mainly expressed in fast muscle fibers, but plays a crucial role in the development of both fast and slow muscle fibers (30). To delve deeper into the specific role of the *MYLPF* gene in muscle development, researchers used homologous recombination to knock out the mouse’s fast skeletal RLC gene (*MYLPF*). Compared to wild-type mice, heterozygous mice showed normal muscle tissue, but homozygous mice exhibited drastically different outcomes. These homozygous mice completely lacked fast and slow muscles, with a high mortality rate shortly after birth, showing a 30% reduction in body weight compared to other groups, and evident deficiency in skeletal muscle formation. These results strongly confirm the critical role of the *MYLPF* gene in the development of fast and slow skeletal muscles (31). This study has uncovered a phenomenon where the expression level of the *MYLPF* gene in the tibialis anterior muscle of Duroc pigs is significantly lower than that in Tibetan fragrant pigs. This differential expression points to a reduction in the level of ubiquitination modification of the protein in Duroc pigs, suggesting that at 30 days of age, the protein possesses fewer ubiquitination modification sites, thereby decreasing its likelihood of being recognized and degraded by the proteasome. Such a change may contribute to maintaining the normal structure and function of muscle fibers, particularly in terms of muscle contraction and energy metabolism. Notably, this gene expression pattern aligns with the characteristic of Duroc pigs’ muscle fibers being rich in glycolytic muscle fibers (fast-muscle fibers), indicating that the muscles of Duroc pigs are more inclined to utilize the glycolytic pathway for rapid energy production. This discovery provides a new perspective for our understanding of the metabolic properties of pig muscle fiber types and their impact on energy supply mechanisms.

The myosin light chain 1 (*MYL1*) gene, as a crucial member of the essential myosin light chain family, plays a pivotal role in muscle biology. The *MYL1* gene encoding two isoforms, MLC1f and MLC3f, which are integral to muscle function (32, 33). The expression of the *MYL1* gene is regulated by various regulatory elements, such as muscle-specific enhancer elements, myocyte enhancer factor 2 (MEF2), and myocyte enhancer factor 3 (MEF3). These regulatory elements ensure that the *MYL1* gene is expressed at the correct time and place, maintaining the normal function of muscle tissue (34). To validate the role of the *MYL1* gene in muscle development, Gianina’s research team constructed a morpholino knockdown model of *MYL1* in zebrafish. The results showed that when the expression of the *MYL1* gene was inhibited, the zebrafish exhibited abnormal muscle development, significantly reduced mobility, and severely damaged muscle fiber tissue. These findings demonstrate the indispensable role of the *MYL1* gene in maintaining the normal formation of muscle fibers and ensuring proper muscle function (35). In the present study, the *MYL1* protein of Duroc pigs contained more abundant ubiquitination modification protein sites compared to that of Tibetan fragrant pigs, a result that may play a key role in facilitating the completion of complex physiological functions in their muscles.

*MYH3* and *MYH7* genes are key factors encoding myosin heavy chain and play an indispensable role in muscle development and function regulation (36–38). The *MYH3* gene is responsible for encoding myosin heavy chain 3, which not only regulates the conversion of muscle fiber types, but also affects fat formation (39). Bharadwaj has previously knocked out the *MYH3* gene in adult mice in a study that showed a reduction in muscle weight and a reduction in muscle fiber size in the mice, increased fibrosis of the muscle, and exhibited structural and functional abnormalities of the skeletal-muscular system (38). On the other hand, *MYH7* (myosin heavy chain 7) responsible for encodes the myosin heavy chain of slow muscle fibers. Furthermore, mutations in the *MYH7* gene can potentially disrupt interactions between myosin and titin with myomesin, M-protein, or sarcomeric structures, leading to Laing Early-Onset Distal Myopathy (MPD1) and tibial muscular dystrophy (40). These findings highlight the critical role of gene mutations in the pathogenesis of muscle diseases, providing valuable theoretical foundations for understanding and treating these conditions. In Cho’s study, *MYH3* was found to be highly co-localized with *MYH7*, a molecular marker for slow muscle fibers, in porcine skeletal muscle (39). In this study, the expression of ubiquitination modification peptides on *MYH3* protein was up-regulated in Duroc pig skeletal muscle, *MYH7* protein showed the presence of multiple modification peptides, and the different peptides showed opposite expression trends. This implies that Duroc pigs have more ubiquitination modification sites on the *MYH3* protein, whereas the *MYH7* protein undergoes a finer and more complex ubiquitination modification process in the fast-twitch-dominated muscle fibers. Different ubiquitination modifications may be involved in the regulation of *MYH7* stability, activity or interaction with other proteins, respectively, to precisely regulate key physiological processes, such as contractile performance, metabolic efficiency and energy utilization in slow-type muscle fibers. In Duroc pigs during rapid growth, a certain number of slow muscle fibers are required to maintain a certain level of muscle endurance and fatigue resistance. However, proteins encoding slow muscle fibers require stable regulation of protein function, so *MYH3* proteins may contain more ubiquitination modification sites to play important roles in muscle fiber type conversion and energy metabolism. *MYH7* proteins may play different physiological functions in organisms, reflecting the diversity and complexity of protein function in organisms. Ubiquitination modification protein, as an Ubiquitination modification, as one of the important means of protein function regulation, may confer different functions to target proteins or affect the activity of proteins and their interactions with other molecules through different types and degrees of ubiquitination modification in response to different states of the organism.

The troponin complex plays a crucial role in the regulation of muscle contraction. The complex contains three subunits: troponin C, troponin I, and troponin T, which work together to ensure the precise regulation and efficient execution of muscle contraction (41). Slow skeletal troponin I (*TNNI1*) is a subunit of troponin that inhibits troponin, and the inhibitory region is highly conserved, but the expression of the protein may be altered during development (42). Mutations in troponin C (Slow skeletal troponin C,Cardiac troponin C,*TNNC1*) mainly cause hypertrophic cardiomyopathy (HCM), and the effect of this gene on skeletal muscle function has not been investigated, and it is also by possible that the skeletal muscle function induced by this gene is disorders are considered to be one of the symptoms of HCM (43). Muscle fibers that predominantly express troponin C2 (*TNNC2*) in postnatal muscle change with age to a 1:1 distribution of slow: fast muscle fibers (44). Jin’s study found that troponin T2 (Cardiac troponin T, *TNNT2*) is expressed in rat and mouse neonatal skeletal muscle and that RNA splicing produces isoform conversion during subsequent development (45). In this study, the expression of *TNNC2* and *TNNT2* was found to be significantly higher in Duroc pigs than in Tibetan fragrant pigs, and correspondingly, the expression of *TNNI1* and *TNNC1* was down-regulated, implying that there were more ubiquitination modification sites for *TNNC2* and *TNNT2* proteins and fewer ubiquitination modification sites for *TNNI1* and *TNNC1* proteins, and that the proteins were more stable, in tibialis anterior muscles in Duroc pigs. This is important for understanding the muscle contraction mechanism. In this complex physiological process, protein degradation and synthesis is a highly coordinated and dynamic process involving multiple factors. We hypothesize that in order to regulate protein function more precisely in response to the shift in muscle fiber type and rapid cell turnover, *TNNC2* and *TNNT2* proteins have achieved the maintenance of a dynamic balance between troponin degradation and synthesis in fast-muscle fiber dominant myofibers through the addition of ubiquitination modification proteins to the site. In contrast, *TNNI1* and *TNNC1* proteins tend to exist more stably in fast myofiber dominant muscle fibers. However, this speculation requires more in-depth studies to fully resolve the underlying molecular mechanisms. Future studies need to explore the specific functions of these ubiquitination modification sites and how they affect troponin stability and activity.

The sarcomere, as the basic contractile unit of skeletal muscle, plays a crucial role in muscle function. Titin (*TTN*) connects the thin filaments (actin) and thick filaments (myosin) of the sarcomere, acting not only as a scaffold and signaling platform but also providing tension and elasticity to the muscle (46, 47). *TTN* is pivotal in initiating and regulating the interaction between actin and myosin, undergoing phosphorylation *in vivo* (48). Research has shown that even isolated *TTN* molecules or skeletal muscle myofibrils experience subtle unfolding-refolding transitions in their immunoglobulin-like (IgC2) domains under physiological force levels. These folding changes, in conjunction with the myosin system, optimize energy utilization during muscle stretching and active contraction (49). Notably, most passive force generated during muscle stretching is contributed by *TTN*. Through alternative splicing of exons, *TTN* can modulate stiffness between different skeletal muscles, further demonstrating its functional diversity and flexibility (50). Within the myotome, the process of myosin turnover and remodeling begins with a highly specific cleavage mediated by calpain, which precisely targets TTN (tropomyosin), actin, and other key protein components of the myosin complex. Subsequently, these cleaved protein fragments produced by UPS are broken down into small molecule peptides and amino acids for reuse in intracellular protein synthesis and repair processes. This process not only ensures the dynamic homeostasis of protein composition within the sarcomere, but is also critical for maintaining the structural integrity, functional adaptability, and regenerative potential of muscle (48). In this study, multiple ubiquitin-modified peptides were found on *TTN* protein, and different peptides showed opposite expression trends. It is speculated that *TTN* proteins may have complex and diverse functional regulatory mechanisms in organisms, and their ubiquitination modification protein may be finely regulated by multiple signaling pathways and physiological states. Therefore, further in-depth studies and explorations are needed to investigate the specific functions and regulatory mechanisms of *TTN* proteins in muscle and other tissues, as well as their relationship with disease development. These studies will help to reveal the basic laws of muscle biology and provide new ideas and methods for the prevention, diagnosis and treatment of related diseases.

Actin, a versatile protein, plays a critical role in various cellular activities, including contraction, migration, division, and signal transduction (51, 52). In the complex biological systems of vertebrates, actin exhibits diverse forms, with at least six actin isoforms identified (53). Among these, β-actins and γ-actins are two widely expressed cytoplasmic actins involved in regulating numerous cellular functions (54). The genes encoding γ-actins, *ACTG1* and *ACTG2*, are indispensable in cellular processes (51). In a detailed study by Bunnell, an intriguing phenomenon was discovered: although γ-actins are not essential during mouse embryonic development, the knockdown of *ACTG1* gene expression significantly impacts embryonic development. This manifests as impaired cell growth and reduced cellular activity, leading to delayed embryonic development and smaller body size. Further research revealed that the underlying mechanism for these defects is an increase in apoptosis levels, which explains the reduced cellular activity (55). This finding not only highlights the crucial role of actin in embryonic development but also provides new perspectives for future research on cell growth, activity, and apoptosis.

In summary, these research findings provide crucial insights into the molecular mechanisms underlying pig skeletal muscle development. Key proteins show differences in ubiquitination levels in different pig breeds in China and the West, and this disparity may cause differences in the total number and type of myofibrils as well as the rate and quality of muscle growth among pig breeds by regulating myofibrillar production, proliferation, differentiation, as well as muscle metabolism and energy utilization, ultimately leading to differences in muscle development and body size. This finding provides new ideas and methods for in-depth study of the molecular mechanisms of muscle development among pig breeds, as well as new targets and directions for pig breed improvement and breeding. The specific mechanisms and effects of the disparity in ubiquitination levels of key proteins between Chinese and Western pig breeds still require further in-depth study.

## 5 Conclusion

In this study, we successfully identified 109 differentially expressed ubiquitinated peptides distributed across multiple cellular compartments such as cytoplasm and nucleus. These ubiquitinated peptides belong to proteins that play roles in key pathways including Glycolysis/Gluconeogenesis and the Hippo signaling pathway. Further analysis revealed differential expression of genes involved in skeletal muscle ubiquitination modification:*MYL1*, *MYH3*, *TNNC2*, *TNNI1*, *MYLPF*, *MYH1*, *MYH7*, *TNNT2*, *TTN*, and *TNNC1*. This research not only provides theoretical foundations for understanding ubiquitin-marked proteins in muscle metabolism across different pig breeds, but also offers important insights for exploring the molecular mechanisms of muscle growth and development.

## Data Availability

The datasets presented in this study can be found in online repositories. The names of the repository/repositories and accession number(s) can be found at: http://www.proteomexchange.org/, PXD051115.

## Supplementary material

SUPPLEMENTARY DATA SHEET 1 Appendix A: Directed Acyclic Graph of GO enrichment of proteins belonging to ubiquitination modification peptide differentially expressed in Duroc pigs and Tibetan fragrant pigs.
