# Supplementary material for: Differential analysis of ubiquitin-proteomics in skeletal muscle of Duroc pigs and Tibetan fragrant pigs
Source: Front Vet Sci. 2024 Aug 30;11:1455338. doi: 10.3389/fvets.2024.1455338 (PMC11395495; doi:10.3389/fvets.2024.1455338)
Supplement: SUPPLEMENTARY DATA SHEET 1 — Appendix A: Directed Acyclic Graph of GO enrichment of proteins belonging to ubiquitination modification peptide differentially expressed in Duroc pigs and Tibetan fragrant pigs. [file Data_Sheet_1.pdf]

Supplementary material

Appendix A Directed Acyclic Graph of GO enrichment of proteins belonging to ubiquitination modification peptide differentially expressed in Duroc pigs and Tibetan fragrant pigs.

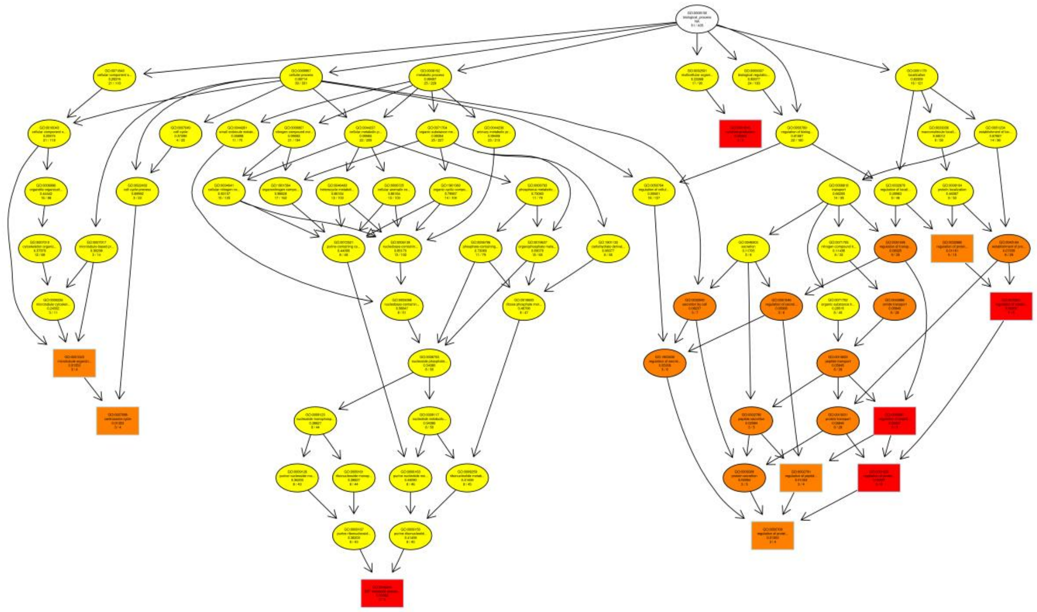

Supplementary Figures 1

GO function enrichment bubble plots under the classification of Biological Process (BP) of Duroc pigs and Tibetan fragrant pigs.

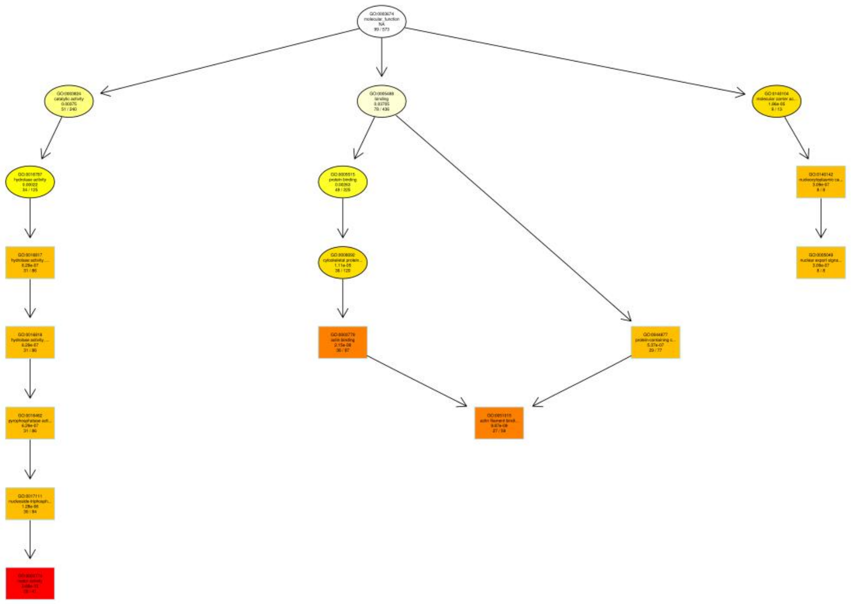

Supplementary Figures 2

GO function enrichment bubble plots under Molecular Function (MF) of Duroc pigs and Tibetan fragrant pigs.

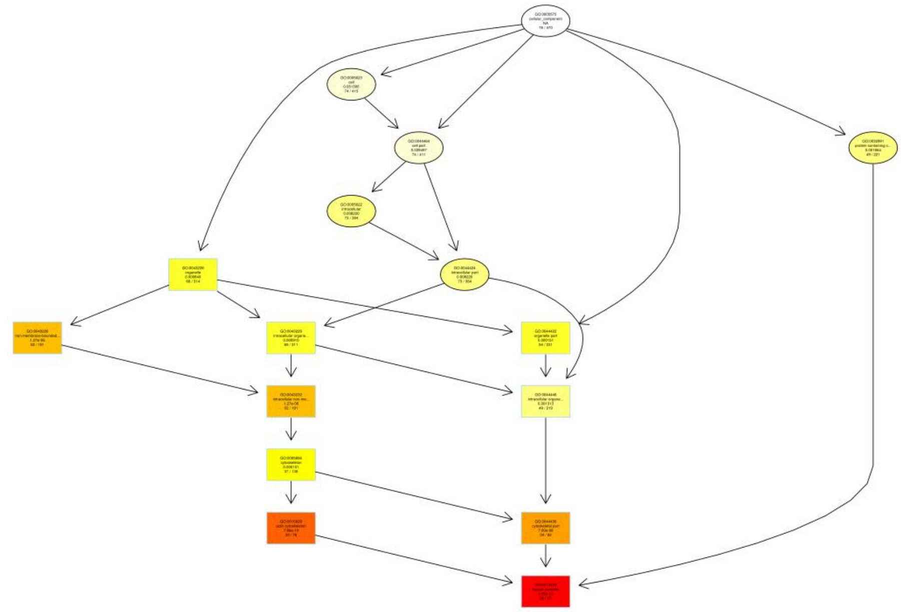

Supplementary Figures 3

GO function enrichment bubble plots under Cellular Component (CC) of Duroc pigs and Tibetan fragrant pigs.
